# Acceptance and Attitude Toward Cosmetic Surgeries in the Western Region of Saudi Arabia: A Cross-Sectional Survey

**DOI:** 10.7759/cureus.45292

**Published:** 2023-09-15

**Authors:** Rawaan S Almajnoni, Mohammed Alharbi, Fahad K. Aljindan., Lina AlSulami, Noura Alsulami, Wael Waiz, Alabbas Alshrif, Hatim Almaghrabi, Abdulaziz Jastania

**Affiliations:** 1 College of Medicine, Umm Al-Qura University, Makkah, SAU; 2 Plastic, Aesthetic, Hand, Burn and Reconstructive Surgery, ‏King Abdullah Medical City, Makkah, SAU; 3 Plastic Surgery, King Abdullah Medical City, Makkah, SAU

**Keywords:** general public, plastic surgery, cosmetic surgery, attitude, acceptance

## Abstract

Cosmetic surgery refers to any surgical intervention that alters normal bodily characteristics in order to attain a more appealing appearance as perceived by the patient. Data from the American Society of Plastic Surgeons (ASPS) indicates a consistent rise in plastic surgery procedures over time. The objective of our research is to carry out a cross-sectional investigation to assess the perceptions and attitudes toward cosmetic surgery among individuals residing in the western region of Saudi Arabia.

This study employed a descriptive, cross-sectional methodology. The intended sample includes residents of Makkah and Medina Regions who are 18 years of age or older, representing the general population. Data collection was carried out through an online questionnaire created using Google Forms, which was disseminated electronically via social media platforms. The questionnaire gathered demographic information and assessed participants' attitudes and acceptance towards cosmetic surgery.

The study included a total of 1249 participants, with 1064 (85%) being female and 185 (15%) being male. In our sample, the overall acceptance rate for cosmetic surgery was 54.2%. There was a significant correlation between the acceptance level and factors such as gender, age, marital status, and occupational status (P value <0.001) for all the aforementioned factors. However, the level of education and financial status were not significantly associated.

The majority of the participants in our study were accepting of cosmetic surgery. However, to gain a more comprehensive understanding of the acceptance of aesthetic surgery in Saudi Arabia, further research should be conducted across the country to assess the attitudes of the wider population.

## Introduction

Cosmetic surgery can be described as any surgical intervention that modifies or enhances normal bodily characteristics, with the aim of achieving a more attractive appearance according to the patient's perception [1,2]. Conversely, surgical interventions aimed at attaining a normal appearance for body features that are abnormal due to trauma, congenital anomalies, developmental irregularities, or illness do not qualify as cosmetic surgery [1].

The American Society of Plastic Surgeons (ASPS) reports that the number of plastic surgery procedures has consistently grown over the years. In 2018, individuals aged between 35 and 50 accounted for the largest proportion of surgical procedures performed [3]. 

A conducted Study in 2019 revealed that Saudi Arabia ranks 29th among the top 30 countries with the highest rates of cosmetic procedures performed worldwide [4]. Given the rise in cosmetic surgery procedures, it is crucial to investigate the underlying factors driving this growing inclination towards such interventions.

The decision and inclination to undergo cosmetic surgery are influenced by various factors, including social networks and epidemiological elements, as well as psychological characteristics such as body image, self-esteem, and other personality traits [5,6]. Furthermore, evolutionary biologists have associated the desire for change with inherent biological drives related to mate selection and reproduction [7].

In the past, the public in Saudi Arabia held negative views toward cosmetic surgery and deemed it unacceptable, primarily due to influential factors such as religious beliefs and the associated health risks of the procedures [8].

A previously published study sought to comprehend the attitudes toward cosmetic surgery among women in China and the Netherlands, across all cultural groups and revealed a favorable attitude toward facial appearance concerns and materialistic beliefs [9]. Age and beauty-ideal internalization were significant positive predictors among Chinese women while body appreciation was a significant positive predictor for both Chinese and Dutch women [9].

A study conducted in Riyadh, Saudi Arabia, in 2019 aimed to evaluate the attitude and acceptance of cosmetic surgery among patients in a tertiary care hospital. The results indicated that 237 (60.9%) patients agreed that cosmetic surgery is beneficial, as it can help individuals feel better about themselves, while 104 (26.8%) patients disagreed with this perspective [8]. A separate study carried out in Majmaah, Saudi Arabia, between 2019 and 2020 found that 47% of the participants expressed approval for cosmetic surgery [10].

Attitudes toward cosmetic surgery may shift over time or vary across different geographical areas. As there has not been a previous study in Western Saudi Arabia specifically focusing on the acceptance of and attitude toward cosmetic surgery, the aim of this research is to explore the acceptance and attitudes toward cosmetic surgery among the general population in the Western region of Saudi Arabia.

## Materials and methods

In this cross-sectional study, we focused on the general population of the western province of Saudi Arabia, including both genders residing in the region, and only excluding those who declined to participate. We determined the sample size to be 384 using Epi Info software Ver 2.1 (CDC, Atlanta, GA) [11]. Following this, we obtained ethical approval from the Biomedical Ethics Committee of Umm Al-Qura University, Makkah City, Saudi Arabia, with approval number HAPO-02-K-012-2023-01-1377. We then distributed our online questionnaire to the target population using a convenience sampling technique and received responses from 1249 participants.

For our study, we utilized a validated survey instrument from a previously published study [8]. We adopted the English version of the questionnaire, which we translated into Arabic by two independent bilingual translators who first translated the English version to Arabic and then performed a backward translation from Arabic to English. This allowed us to compare the two English versions and make adjustments to the Arabic version accordingly. To ensure the clarity and reliability of our survey, we conducted a pretest with 20% of our sample size, excluding their data from the study's results.

The questionnaire comprised two sections. The first collected sociodemographic data from the participants while the second assessed their acceptance of and attitudes toward cosmetic surgeries. The second section contained 15 items divided into three subscales: intrapersonal, social, and consideration, with five questions representing each subscale. Items were rated on a seven-point Likert scale, ranging from 1 (strongly disagree) to 7 (strongly agree); higher scores indicated more positive attitudes toward cosmetic surgery. A consent form was also attached at the beginning of the questionnaire.

Data were collected and cleaned using an Excel sheet (Microsoft Corporation, Redmond, WA) and analyzed with SPSS software (IBM Corp., Armony, NY). Descriptive analysis employed frequency and percentages to describe categorical data. The one-way analysis of variance (ANOVA) test was used to determine the relationship between different sociodemographic factors and the overall level of acceptance towards plastic surgery.

## Results

This study included a total of 1,249 respondents. There was a higher representation of females, with 1,064 (85%) female and 185 (15%) male participants. The most represented age group was 18-25 years old, comprising 789 (63%) of participants. Most of the sample had higher education, with 874 (70%) holding bachelor's degrees. The majority of respondents were single, with 887 (71%) reporting single status. More details about the sociodemographic information are provided in Table 1.

**Table 1:** Socio-demographic characteristics of study participants

Characteristic		Frequency	Percentage
Gender	Male	185	15%
	Female	1064	85%
Age	<18	74	6%
	18-25	789	63%
	26-30	168	14%
	>30	218	17%
City of residence	Makkah Region	936	75%
	Medina Region	313	25%
Level of education	Highschool education	326	26%
	Bachelor’s degree	874	70%
	Higher education	49	4%
Marital status	Married	317	25%
	Divorced	38	3%
	Widow/er	7	0.6%
	Single	887	71%
Occupational status	Student	731	58%
	Employed	232	19%
	Non-Employed	189	15%
	Others	97	8%
Financial status (monthly income of the family)	Less than 10,000 SR	406	33%
	10,000 – 19,000 SR	638	51%
	More than 19,000 SR	205	16%

The overall acceptance of cosmetic surgery in our sample was 54.2%, indicating nearly equal rates of approval and refusal. This divide was seen across some aspects of the intrapersonal, social, and consideration subscales. For the intrapersonal subscale, statements like "Cosmetic surgery is a good thing because it can help people feel better about themselves" had 220 (17.6%) participants strongly disagreeing and 213 (17.1%) strongly agreeing.

Participants were less divided on the social scale, as most were unwilling to have cosmetic surgery for significant others, careers, or maintaining a youthful appearance. A detailed breakdown of answers is shown in Table 2.

**Table 2:** Questionnaire responses to first-scale items (n=1249)

Statement	Strongly disagree	Disagree somewhat	Disagree a little	Neutral	Agree a little	Agree somewhat	Strongly agree
It makes sense to have minor cosmetic surgery rather than spending years feeling bad about the way you look.	248 (19.9%)	62 (5.0%)	91 (7.3%)	233 (18.7%)	157 (12.6%)	231 (18.5%)	227 (18.2%)
Cosmetic surgery is a good thing because it can help people feel better about themselves.	220 (17.6%)	73 (5.8%)	79 (6.3%)	217 (17.4%)	224 (17.9%)	223 (17.9%)	213 (17.1%)
In the future, I could end up having some kind of cosmetic surgery.	325 (26.0%)	80 (6.4%)	79 (6.3%)	195 (15.6%)	222 (17.8%)	176 (14.1%)	172 (13.8%)
People who are very unhappy with their physical appearance should consider cosmetic surgery as one option.	369 (29.5%)	107 (8.6%)	119 (9.5%)	211 (16.9%)	189 (15.1%)	140 (11.2%)	114 (9.1%)
If cosmetic surgery can make someone happier with the way they look, then they should try it.	252 (20.2%)	92 (7.4%)	85 (6.8%)	258 (20.7%)	223 (17.9%)	159 (12.7%)	180 (14.4%)
If I could have a surgical procedure done for free, I would consider trying cosmetic surgery.	381 (30.5%)	84 (6.7%)	83 (6.6%)	219 (17.5%)	171 (13.7%)	125 (10.0%)	186 (14.9%)
If I knew there would be no negative side effects or pain, I would like to try cosmetic surgery.	337 (27.0%)	89 (7.1%)	75 (6.0%)	190 (15.2%)	185 (14.8%)	155 (12.4%)	218 (17.5%)
I have sometimes thought about having cosmetic surgery.	353 (28.3%)	95 (7.6%)	85 (6.8%)	159 (12.7%)	192 (15.4%)	178 (14.3%)	187 (15.0%)
I would seriously consider having cosmetic surgery, if my partner thought it was a good idea.	543 (43.5%)	85 (6.8%)	93 (7.4%)	207 (16.6%)	149 (11.9%)	92 (7.4%)	80 (6.4%)
I would never have any kind of plastic surgery.	110 (8.8%)	71 (5.7%)	107 (8.6%)	301 (24.1%)	178 (14.3%)	129 (10.3%)	353 (28.3%)
I would think about having cosmetic surgery to keep looking young.	356 (28.5%)	80 (6.4%)	99 (7.9%)	242 (19.4%)	198 (15.9%)	144 (11.5%)	130 (10.4%)
If it would benefit my career, I would think about having plastic surgery.	457 (36.6%)	88 (7.0%)	96 (7.7%)	242 (19.4%)	160 (12.8%)	106 (8.5%)	100 (8.0%)
Cosmetic surgery can be a big benefit to people’s self‑image.	219 (17.5%)	64 (5.1%)	82 (6.6%)	251 (20.1%)	257 (20.6%)	181 (14.5%)	195 (15.6%)
I might seriously consider having cosmetic surgery, if my partner would find me more attractive.	464 (37.1%)	102 (8.2%)	91 (7.3%)	216 (17.3%)	157 (12.6%)	102 (8.2%)	117 (9.4%)
If a simple cosmetic surgery procedure would make me more attractive to others, I would think about having it done.	413 (33.1%)	96 (7.7%)	94 (7.5%)	221 (17.7%)	158 (12.7%)	122 (9.8%)	145 (11.6%)

Tables 3-4 display the responses regarding previous plastic surgery and willingness to have plastic surgery in the future. A total of 777 (62.2%) respondents reported no previous surgeries. The most common procedure was laser surgery, chosen by 358 (28.7%). Laser surgery was also the most popular choice for future surgeries, selected by 569 (45.6%), followed by fillers at 340 (27.2%), and Botox at 271 (21.7%). Conversely, 431 (34.5%) participants expressed no plans to undergo any type of plastic surgery.

**Table 3:** Questionnaire responses to items in previous surgeries (n=1249)

Surgery	Frequency	Percentage
Liposuction	55	4.4%
Breast augmentation	24	1.9%
Hair transplant	17	1.4%
Blepharoplasty	27	2.2%
Filler	113	9.0%
Botox	66	5.3%
Laser hair removal	358	28.7%
Rhinoplasty	26	2.1%
Nefertiti Lift	21	1.7%
Body contouring	18	1.4%
Facelift	18	1.4%
Septoplasty	25	2.0%
Burn reconstructive surgery	29	2.3%
Chin augmentation	17	1.4%
Scar revision surgery	45	3.6%
Tummy tuck	21	1.7%
Tattoo removal	14	1.1%
Other surgeries	40	3.2%
None	777	62.2%

**Table 4:** Questionnaire responses to items in future surgeries (n=1249)

Surgery	Frequency	Percentage
Liposuction	177	14.2%
Breast augmentation	77	6.2%
Hair transplant	109	8.7%
Blepharoplasty	87	7.0%
Filler	340	27.2%
Botox	271	21.7%
Laser hair removal	569	45.6%
Rhinoplasty	197	15.8%
Nefertiti lift	162	13.0%
Body contouring	145	11.6%
Facelift	135	10.8%
Septoplasty	98	7.8%
Burn reconstructive surgery	135	10.8%
Chin augmentation	110	8.8%
Scar revision surgery	183	14.7%
Tummy tuck	176	14.1%
Tattoo removal	32	2.6%
Other surgeries	72	5.8%
None	431	34.5%

Our results also show a significant association between the level of acceptance and gender, age, marital status, and occupational status (P-value is <0.001) for all mentioned factors. The level of education and financial status were not significantly associated. More information is shown in Table 5.

**Table 5:** Analysis of the acceptance of cosmetic surgery * P < 0.05 (significant)

Characteristic	Description	Score	P value
Gender	Male	54.4 ± 25.5	<0.001*
	Female	57.3 ± 24.0	
Age	<18	62.8 ± 19.8	<0.001*
	18-25	52.9 ± 24.1	
	26-30	61.3 ± 22.4	
	>30	65.9 ± 24.5	
Level of education	Highschool education	57.1 ± 24.0	0.367
	Bachelor’s degree	56.6 ± 24.3	
	Higher education	61.6 ± 24.6	
Marital status	Married	64.8 ± 23.2	<0.001*
	Divorced, Widow/widower	67.0 ± 26.9	
	Single	53 ± 23.7	
Occupational status	Student	53.3 ± 23.8	<0.001*
	Employed	62.5 ± 25.5	
	Non-Employed	60.0 ± 23.2	
	Others	65.2 ± 21.7	
Financial status	Less than 10,000 SR	58.3 ± 24.6	0.088
	10,000 – 19,000 SR	55.9 ± 24.1	
	More than 19,000 SR	54.6 ± 23.4	

## Discussion

According to the latest global survey from the International Society of Aesthetic Plastic Surgery (ISAPS), cosmetic surgery has become increasingly prevalent over the last four years among both genders worldwide [12]. In 2019, Saudi Arabia ranked 29 among the countries with the highest rates of cosmetic procedures and the top 30 countries with the highest number of plastic surgeons [4,10].

Previously, Saudi society was largely against cosmetic surgery due to factors like religious beliefs and health risks [8]. However, recent improvements in cosmetic surgery outcomes and the growing use of social networks have changed many concepts of cosmetic surgery [8]. This study aimed to assess the attitude and acceptance of cosmetic surgery among the general population in Western Saudi Arabia. We observed that 54.2% of respondents accepted cosmetic surgery; in contrast, a study conducted in Riyadh, Saudi Arabia, showed that 53.9% of respondents did accept cosmetic surgery [8]. This was dissimilar to a study conducted among female students at Taif University, Saudi Arabia, which showed that the majority of participants (72.3) accepted cosmetic surgery [13]. These differences may be influenced by factors such as mass media, culture, relatives, and religious beliefs [14,15].

For years, the public has modeled clothing, hairstyles, and body types after celebrities, and studies have shown that media influences, such as TV shows, magazines, and movies, can affect body image satisfaction, self-esteem, and the decision to choose cosmetic surgery to change one's appearance [5,16]. According to our data, females had a significantly higher total Acceptance of Cosmetic Surgery Scale (ACSS) score than males, similar to the Henderson-King study [17]. Moreover, 52.2% of our respondents believe that cosmetic surgery is good because it can help people feel better about themselves, in agreement with the Riyadh and Majmaah studies [8,10].

Regarding age groups, our results showed that older adults above 30 years had a higher total ACSS than other age groups [17]. In this study, 62.2% of participants had never undergone plastic surgery before, while the Riyadh, Saudi Arabia, study found that none of the participants had cosmetic surgery [8]. In accordance with our research, a study conducted in Nigeria in 2016 among basic sciences students found that a very small number of students have had or know someone who did these surgeries [15]. These facts might be influenced by religious beliefs and health risks; as in the Nigerian study, 91% of respondents believed that cosmetic surgery is harmful [15]. Our results showed that women were more likely than men to consider having cosmetic surgery, which is consistent with previous research [18].

However, these results need replication with different demographic groups and in different settings to assess whether the acceptance of cosmetic surgery is changing or not.

Strengths and limitations

The present study is the first to examine attitudes toward and acceptance of cosmetic surgery among the population in Western Saudi Arabia, featuring a large sample size. However, there were several limitations. First, the study had a limited number of male participants with higher education. Second, the findings from this specific context may not be easily generalizable to the broader Saudi population.

## Conclusions

The objective of our study was to evaluate the attitude and acceptance of cosmetic surgery among the general population in the Western province of Saudi Arabia. Our findings have important implications for understanding the cultural perspective on cosmetic surgery and provide insights into the current and future interests in cosmetic procedures within the country. Notably, significant variations in acceptance were observed among different age groups, genders, as well as marital and occupational statuses. However, further research is needed across Saudi Arabia to better understand the acceptance of aesthetic surgery within the broader population.
